# Peri‐implant marginal bone loss and systemic statin use: A retrospective cohort pilot study

**DOI:** 10.1002/cre2.509

**Published:** 2021-10-29

**Authors:** Behzad Bahrami‐Hessari, Leif Jansson

**Affiliations:** ^1^ Folktandvården Stockholms län AB Folktandvården Skärholmen Stockholm Sweden; ^2^ Department of Periodontology, Folktandvården Stockholms län AB Folktandvården Eastmaninstitutet Stockholm Sweden; ^3^ Division of Periodontology, Department of Dental Medicine Karolinska Institutet Stockholm Sweden

**Keywords:** bone‐loss, peri‐implantitis, severity, statins

## Abstract

**Objectives:**

The aim was to analyze clinical parameters of peri‐implantitis in human subjects exposed and non‐exposed to use of systemic statins.

**Material and methods:**

This retrospective cohort pilot study compared patient records of 60 exposed individuals to 196 non‐exposed individuals as of 2011 throughout 2017. Source of records were specialist and general dentistry clinics in Public Dental Service, Stockholm County, Sweden. Extent/severity of peri‐implantitis and peri‐implant bone loss were registered as well as intake of systemic statins. Background variables considered were bleeding on probing, bone‐loss, age, gender, earlier periodontitis, prosthetic quality, and smoking. Stepwise linear and logistic regression analysis at the individual level was adopted in order to study the influence of statin use on the severity of peri‐implantitis and the incidence of peri‐implant bone loss. Results were considered statistically significant at *p* < 0.05.

**Results:**

Peri‐implant bone loss was significantly correlated to use of statin after compensation for age and sex.

**Conclusions:**

The results render an actual effect of statins on peri‐implant bone loss plausible. Further research is warranted.

## 1 INTRODUCTION

With the advent of implants a new problem has emerged, namely peri‐implantitis, an inflammatory tissue destruction around dental implants in function (Berglundh et al., 2018; Zitzmann & Berglundh, 2008). The clinical manifestations such as bleeding or suppuration on probing and bone loss are similar to that of periodontitis although differences on microscopic level exist (Berglundh et al., 2011; Carcuac & Berglundh, 2014). The prevalence seems to vary depending on case definition, suggested as between 14% and 30% (Derks & Tomasi, 2015) and reported as up to 45% (Derks et al., 2016). The prevalence is estimated through meta‐analysis to approximately 20% of subjects and 10% of implants, as weighted mean, globally (Lee et al., 2017). This would translate to many patients on a global scale.

Some studies point towards positive effects of 3‐hydroxy‐3‐methyl‐glutaryl‐coenzyme A reductase inhibitor (HMGCR), also known as statin, on the treatment of periodontitis (Lindy et al., 2008; Muniz et al., 2018; Petit et al., 2019). Statins, the effective substance in blood cholesterol lowering medicines, demonstrate antibacterial, anti‐inflammatory and bone‐promoting properties (Bertl et al., 2018; Petit et al., 2019). These properties are all of interest when infections around dental implants are handled.

Systemically used statins have indicated positive effects on the healing of apical periodontitis (Alghofaily et al., 2018; Lin et al., 2009). There should therefore be grounds for believing in the same expected effect on other parts of the periodontal ligament should pathogen elimination be reached. However difficult in the case of periodontitis, it may be easier to adequately eliminate and block infectious agents in certain peri‐implantitis cases where removal of prosthetic and complete closure of the flaps over the implant is possible.

Although above‐mentioned studies on statins suggest a positive effect on periodontally damaged teeth, the overall results also seem inconclusive (Bertl et al., 2017; Saver et al., 2007; Saxlin et al., 2009). Therefore, further investigation on the effect of statins on periodontitis and peri‐implantitis is of interest. This is especially true for peri‐implantitis since the periodontitis and peri‐implantitis lesions differ, regarding cellular composition and lesion anatomy (Berglundh et al., 2011; Carcuac & Berglundh, 2014; Thorbert‐Mros et al., 2015). Thus, use of research outcome regarding statins' effect on periodontitis may not be automatically applicable to peri‐implantitis. Since studies suggest no positive effect of statins on regeneration of periodontal ligament or cementum (Bertl et al., 2017; Bertl et al., 2018) they become interesting as locally used adjunct to peri‐implantitis treatment.

Current state of research seems to show a knowledge gap regarding the impact of statins on the hallmarks of peri‐implantitis in humans. The aim of this study was to analyze clinical parameters of peri‐implantitis in human subjects exposed and non‐exposed to use of systemic statins.

## 2 MATERIALS AND METHODS

### 2.1 Ethical statements

The declaration of Helsinki was adhered to (World Medical, 2013). The ethical approval (DNR: 2019‐04221) was granted by the regional ethics committee of Stockholm County, Sweden. Guidelines on ‘STrengthening the Reporting of OBservational studies in Epidemiology’ (STROBE) were followed (Vandenbroucke et al., 2007). Power calculation with an anticipated difference between the two groups according to presence of peri‐implantitis = 15%, significance level = 0.05 and power = 0.80 shows need of 76 individuals in the exposed group and 228 in the non‐exposed.

### 2.2 Study design and study population

The project was conducted as a retrospective cohort study using data from five randomly selected general dentistry clinics and two departments of a specialist clinic (periodontology and prosthodontics) belonging to Public Dental Service (Folktandvården) of Stockholm, Sweden.

The inclusion criteria were: Implants placed as of the year 2011 and throughout 2017; age 60 years and above; adequate past dental record available including periodontal and medical history; adequate radiographs available of the implants at baseline (radiographs taken in connection with prosthetic loading) and at follow‐up at least 1‐year post surgery.

The exclusion criteria were uncontrolled diabetes; smoking 10 or more cigarettes per day; lost implants for unknown or other reasons than peri‐implantitis.

**Figure 1:**
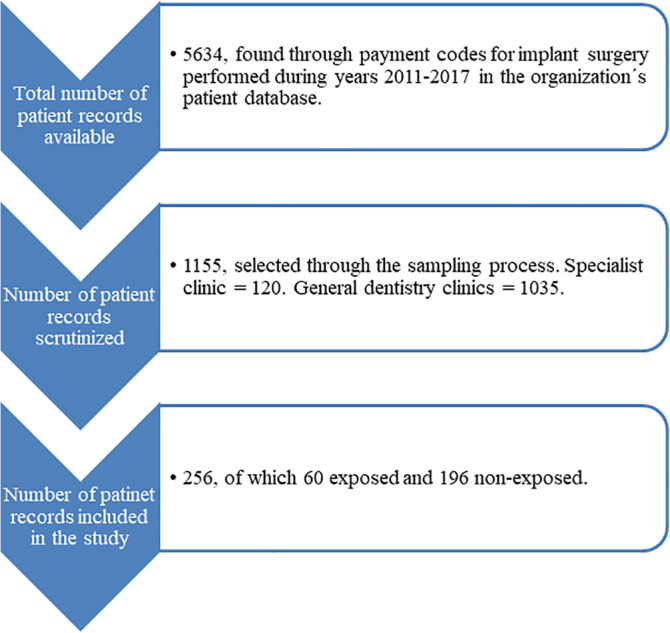
Flow chart

The first step was to identify the patients with implant treatment 2011–2017 at age ≥60 years (See flow chart in Figure 1). This resulted in 5634 available patient records. The next step was a systematic sampling process randomly selecting 1155 patient records (10% from specialist clinics and 90% from general dentistry clinics) to scrutinize according to inclusion and exclusion criteria. By the end of the data collection phase in late 2019, final number of patient records included for statistical analysis was 256. Due to the relative rarity of those exposed, the ratio of the two groups in the study was 1:3 with 60 exposed and 196 non‐exposed individuals.

### 2.3 Variables

The following variables were collected from the patient records after inclusion:

Use of systemic statins; Age (years); Gender; Smoking <10 cigarettes per day at time of surgery and thereafter; History of periodontitis/peri‐implantitis; Recurrent treatment or supportive therapy at least once a year during the study period; Number of implants; Bleeding/suppuration on probing recorded during the study period; Peri‐implant bone loss: >3 mm initial remodeling compared to bone level at baseline (radiographs taken in connection with prosthetic loading); Peri‐implantitis: bleeding/suppuration on probing and Peri‐implant bone loss; Severity of peri‐implantitis: the distance (rounded to nearest millimeter) beyond 3 mm initial remodeling for the most severe site affected by bone loss (whole millimeters); Extent of peri‐implantitis: number of implants with peri‐implant bone loss; Prosthetic risk: If the distance on the baseline radiograph between crown‐abutment junction and the crestal bone was shorter than 3 mm or implant placed closer than 1 mm to neighboring teeth.

Extent and severity of peri‐implantitis were measured in line with the case definition described in guidelines of world periodontology workshop in 2017 (Caton et al., 2018). Loss of attachment or supporting bone was analyzed by studying radiographs at least 1‐year post surgery and measuring for loss greater than the initial remodeling of 3 mm, rounded to nearest millimeter for bone loss, compared with baseline radiographs.

### 2.4 Radiography measurement protocol

For all measurements and viewing of radiography, a dental imaging software was used (Planmeca Romexis version 5.3.4.39, Helsinki, Finland). Whenever possible the measurements were conducted on bitewing or apical radiographs with minimal distortion of implant threads. Measurements on panoramic radiograph was restricted to bone loss measurement only and used only when intraoral radiographs of adequate quality were not available (Riecke et al., 2015).

Baseline radiograph from first prosthetic loading was compared to radiograph at least 1‐year post surgery for information on eventual bone remodeling. The period in between the two radiographs was considered initial remodeling phase. Measurements were made on both radiographs. When radiographs on later dates were available, this measurement protocol was undertaken for the radiograph with adequate quality of latest date. Baseline radiograph was used to assess the initial relationship of the prosthetic to peri‐implant bone. Radiographs at 1‐year post surgery and later were used to assess eventual bone loss beyond an initial bone remodeling.

The software's measurement tool was calibrated on each radiograph by known diameter of the implant, taken from the patient record. The crown‐abutment junction was used as starting point to treat measurement of soft tissue thickness on all different types of implants alike. The distance between crown‐abutment junction and bone was measured for an anticipated soft tissue thickness of 3 mm. This threshold and the starting point of measurement were chosen due to current lack of exact knowledge regarding an initial remodeling around healthy implants in humans and the possible effect of prosthetics on peri‐implant tissues (A. Lee et al., 2011; Linkevicius & Apse, 2008; Spinato et al., 2019; Yi et al., 2020). Therefore, any distance measuring between 2.5 mm and 3.4 mm on the radiographs was considered as 3 mm initial remodeling. On the baseline radiograph, if the mentioned distance was shorter than 3 mm or implant placed closer than 1 mm to neighboring teeth it was defined in this study as a ‘prosthetic risk’. On the 1‐year or later radiographs, if bone loss was larger than the 3 mm line, measured from the starting point, it was defined as ‘peri‐implant bone loss larger than 3 mm initial remodeling’ regardless of the reason for that bone loss. The remaining bone loss beyond the 3 mm line was considered as severity of bone loss potentially due to peri‐implantitis. Any measurement beyond 3 mm initial remodeling, yet smaller than 0.5 mm was rounded down. Any bone loss of 0.5 mm or larger was rounded up. All severity measurements were rounded to nearest millimeter. The most severe site on the most severely affected implant was recorded as ‘severity’. The total number of affected implants with any bone loss beyond the 3 mm line were defined as ‘extent’.

### 2.5 Statistical methods

Descriptive statistics and statistical analyses were performed using a statistical package (IBM SPSS Statistics 26.0; SPSS Inc.). In all analyses, the statistical computational unit was at subject level. Chi‐square analyses or Fischer's exact test were used in order to analyze differences between groups according to categorical variables, while *t*‐tests were used for numerical variables. Pearson correlations were calculated for pair‐wise comparisons between variables. Stepwise linear and logistic regression analysis at the individual level was adopted in order to study the influence of statin use on the severity of peri‐implantitis and the incidence of peri‐implant bone loss. The regression analyses included investigated independent variables as potential confounders. Variables with significance level *p* < 0.05 in the last step after compensation for age and sex were included in the model. Results were considered statistically significant at *p* < 0.05.

## 3 RESULTS

The intra‐examiner reliability was estimated by rerecording the variables bleeding on probing, peri‐implant bone loss, extent and severity from randomly selected records of 20 patients included in the study. The reliability was analyzed through Cohen's Kappa statistic with the consistency for measurement registrations at 100% (*κ* = 1).

**Figure 2:**
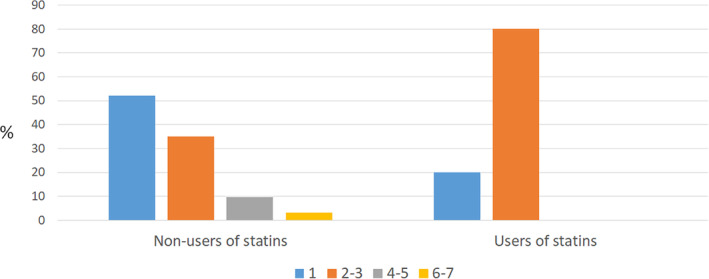
Subject level distribution of number of implants with peri‐implantitis based on statin use

Description of investigated variables stratified according to use of statins is presented in Table 1. The average follow‐up time was 4.0 years (SD 1.9). The cohort split in two groups, exposed and non‐exposed, involved 256 subjects in total. The exposed group contained 60 individuals while the non‐exposed group 196. The exposed individuals were significantly older (*p* = 0.001) and significantly more males (*p* = 0.03) compared to the non‐exposed. In addition, peri‐implant bone loss >3 mm initial remodeling was significantly more frequent among the non‐exposed (*p* = 0.03) as was the severity of peri‐implantitis (*p* = 0.02). The distribution of number of implants was approximately equal for exposed and non‐exposed groups, while the distribution of number of implants with peri‐implantitis differed between the exposed and non‐exposed groups (Figure 2).

**Table 1:** Description of investigated variables according to statin use

Variable	*N* (use of systemic statin [no/yes])	Use of systemic statin		*p*
		No	Yes	
Age (mean [SD])	196/60	72 (7.4)	77 (8.8)	0.001
Sex (% females/% males)	196/60	52/48	35/65	0.03
Smoking <10 cigarettes per day at and after surgery (%)	169/49	18	8.2	NS
Prosthetic risk (%)	195/55	17	16	NS
History of periodontitis or peri‐implantitis (%)	196/55	49	58	NS
Recurrent periodontal treatment (%)	171/39	56	62	NS
Number of implants (mean [SD])	196/55	2.5 (1.5)	2.3 (1.4)	NS
Peri‐implant bone loss >3 mm initial remodeling (%)	194/54	28	13	0.03
Bleeding on probing on at least one implant (%)	179/43	32	44	NS
Peri‐implantitis (%)	191/54	16	9.3	NS
Extent of peri‐implantitis (mean [SD])	191/54	0.36 (1.0)	0.19 (0.62)	NS
Severity of peri‐implantitis (mean [SD])	192/54	0.56 (1.4)	0.20 (0.79)	0.02

*Note*: Chi‐square analyses, Fischer's exact test or *t*‐tests. Significance level *p* < 0.05.

History of periodontitis/peri‐implantitis was positively correlated to the severity of periodontitis (*r* = 0.15, *p* = 0.02, Table 2) and significantly more frequent for peri‐implant bone loss >3 mm initial remodeling (73%, *p* < 0.001, Table 3) compared to 46% for those without peri‐implantitis (Table 3). Prosthetic risk was significantly associated to severity of peri‐implantitis (*r* = 0.14, *p* = 0.03, Table 2) and significantly more frequent for implants with bone loss >3 mm initial remodeling (27%, *p* = 0.02, Table 3).

**Table 2:** Bivariate correlations (*r*) between ‘severity of peri‐implantitis’ and investigated variables

Variable	*N*	Severity of peri‐implantitis	*p*
Age	249	0.01	NS
Sex (female = 0, male = 1)	249	−0.03	NS
Smoking <10 cigarettes per day at and after surgery	216	0.09	NS
Peri‐implant bone loss >3 mm initial remodeling	249	0.66	<0.001
History of periodontitis or peri‐implantitis	248	0.15	0.02
Recurrent periodontal treatment	211	0.07	NS
Prosthetic risk	249	0.14	0.03

*Note*: Pearson correlation coefficients. Significance level *p* < 0.05.

**Table 3:** Description of investigated variables stratified according to ‘peri‐implant bone loss >3 mm initial remodeling’

Variable	*N*	Peri‐implant bone loss >3 mm initial remodeling		*p*
		+	−	
Age (mean [SD])	258	73.1 (7.4)	73.1 (7.8)	NS
Sex (females/males)	258	55%/45%	46%/54%	NS
Smoking <10 cigarettes/day at and after surgery (%)	221	20	14	NS
Bleeding on probing on at least one implant (%)	225	40	33	NS
History of periodontitis or peri‐implantitis (%)	252	73	46	<0.001
Prosthetic risk (%)	251	27	14	0.02
Recurrent periodontal treatment (%)	211	59	56	NS

*Note*: Chi‐square analyses, Fischer's exact test or *t*‐tests. Significance level *p* < 0.05.

The degree of peri‐implantitis was significantly more severe for the non‐exposed group in the bivariate comparison (Table 1). However, in the last step of the multiple linear regression analysis after compensation for age and sex, a significant association between severity of peri‐implantitis and statin use was not found, albeit close (*p* = 0.08, Table 4a). In the multiple logistic regression analysis using peri‐implant bone loss >3 mm initial remodeling as dependent variable, use of statin was significantly correlated to that variable after compensation for age and sex (*p* = 0.038, Table 4b). None of the investigated independent variables in Table 1 were included as significant (*p* < 0.05) confounders in the last step of the regression analyses.

**Table 4:** Results of multiple regression analysis using ‘severity of peri‐implantitis’ (A: linear) and ‘peri‐implant bone loss >3 mm initial remodeling’ (B: logistic) as dependent variable

A. *N* = 246			
Independent variables	Beta	SE	*p*
Age (years)	0.003	0.011	0.76
Sex (female = 0, male = 1)	−0.02	0.17	0.91
Use of statins (no = 0, yes = 1)	−0.36	0.21	0.08

B. *N* = 248			
Independent variables	Odds ratio	Confidence interval	*p*
Age (years)	1.00	(0.96; 1.04)	0.94
Sex (female = 0, male = 1)	0.80	(0.45; 1.45)	0.47
Use of statins (no = 0, yes = 1)	0.40	(0.17; 0.95)	0.038

## 4 DISCUSSION

This study showed a significant correlation between systemic statin use and peri‐implant bone loss, while a non‐significant correlation between statin use and severity of peri‐implantitis was found (*p* = 0.08). Moreover, this study demonstrated that peri‐implant bone loss and severity of peri‐implantitis were significantly correlated to prosthetic risk using bivariate analyses. Other experimental animal studies have also suggested similar positive effect of statin use on implants (Moriyama et al., 2008; Moriyama et al., 2010), albeit inconclusive (Li et al., 2016; Ma et al., 2008). Additionally, history of periodontitis/peri‐implantitis was in this study found as significantly correlated to severity of peri‐implantitis, as indicated before (Chrcanovic, 2017; Corcuera‐Flores et al., 2016; Kandasamy et al., 2018; Ting et al., 2018; Turri et al., 2016).

Although statin use was significantly correlated with peri‐implant bone loss, a correlation of only borderline significance between statin use and severity of peri‐implantitis was found when adjusted for gender and age. This result could be an effect of patients with peri‐implant bone loss >3 mm initial remodeling in the non‐exposed group, who may have lacked the bleeding or suppuration requirements in their records for diagnosis of peri‐implantitis. Since the non‐exposed group showed a larger discrepancy between the level of peri‐implant bone loss and the number of patients diagnosed with peri‐implantitis in comparison with the exposed group, the lack of accurate bleeding or suppuration record is suspected as a source of bias. It is seldom older patients use systemic statins due to, or in combination with, other medication for diabetes, high blood pressure or cardiovascular diseases, where higher rates of bleeding on probing may occur. If so, the found discrepancy act as an additional risk indicator for peri‐implantitis in the exposed group (Ting et al., 2018; Turri et al., 2016).

Former smokers are in this study included in the non‐smoker category, albeit with an uncertain and perhaps diluting effect on the hallmarks of peri‐implantitis. Yet, the exposed group showed less peri‐implant bone loss. Moreover, the non‐exposed group seem to contain more smokers than the exposed group, albeit not on a significant level. Therefore, suspicion about the records of the exposed and non‐exposed groups not showing the correct bleeding on probing may be valid, hence the peri‐implantitis results.

The peri‐implant bone loss may be the effect of other factors than just ongoing peri‐implantitis. Nevertheless, such factors when randomly occurring will be found almost similarly in all groups. In this study, prosthetic risk as a risk indicator for peri‐implant bone loss was found almost equally in exposed as the non‐exposed group. As an example, the prosthetic risk shows the expected randomization effect occurring in this study as expected. Thus, peri‐implantitis remains one of the most probable causes for this bone loss.

Prosthetic risk was correlated with peri‐implant bone loss as well as with severity of peri‐implantitis as shown in other studies (Serino & Hultin, 2019; Yi et al., 2020). These findings may add to the strength of the correlation of statin use with peri‐implant bone loss simply because the latter is a part in the diagnosis of peri‐implantitis and a result of it.

Several findings add to the suspicion that the information on bleeding or suppuration in the patient records may be a source of bias. First, considering the randomization effect in the distribution of implants between the groups and the two variables number of implants and prosthetic risk. Second, bearing in mind a found close correlation between use of systemic statins and severity of peri‐implantitis. Third, in this study various clinicians' measurement protocols regarding bleeding on probing is unknown, while a known bone loss measurement protocol is used on radiographs unchanged over the course of years. The statistically significant findings on peri‐implant bone loss >3 mm initial remodeling may therefore be closer to the reality of peri‐implantitis than the lack of significant correlation between statin use and severity of peri‐implantitis. Additionally, lack of significant correlation between statin use and extent/severity of peri‐implantitis may partly be due to time on medication, doses or types of statins. These were not variables in this study, as they were not consistently specified in patients' records. However, variations in type and dose of systemic statin use have previously shown marginal effect (Bertl et al., 2017).

The final size of this study is different from the ideal size and may have had an impact on the outcome. Although random occurrence of the variable prosthetic risk and number of implants among the groups show the study being of adequate size concerning a randomization effect. In addition, detailed information on variables such as distribution of diabetes patients between and within the groups of the cohort would have been of interest.

There are some strengths and limitations with the current study. The goal was to minimize selection bias mainly by randomization through several methods. The methods were systematic sampling of all participants in the order they attended treatment; stratification by age; selection from several clinics; selection over the course of a relatively long time (“Bias in Cohort Studies,” Hill & Kleinbaum, 2005; Sedgwick, 2014b). This approach may have reduced effects of confounding and selection bias (Sedgwick, 2014a). However, a relatively small number of participants for analysis of a certain rare variable may be a source of bias if an expected randomization effect does not occur (Sedgwick, 2014c). In addition, bias may occur in areas where measurement methodologies are difficult to predefine, such as measurement on radiographs depending on the angle of x‐ray in this study or occasional use of panoramic radiograph (Riecke et al., 2015). Considering clinical data, relying on historical measurements by various clinicians with unknown measurement protocols may have raised generalizability. However, it may also have created uncertainty about validation of measurements, such as bleeding on probing as information bias (Salkind, 2010). Additionally, rounding the measurements to whole millimeters may have increased bias about very small tissue destructions (Lagunov, Sun, & George, 2019).

### 4.1 Conclusion and future direction

In conclusion, a significant negative correlation between statin use and peri‐implant bone loss >3 mm initial remodeling was found. Within the limits of our study, this finding renders an actual effect of statins on peri‐implant bone loss plausible and on severity of peri‐implantitis possible.

Since no other studies have yet been published considering the effect of statins on the hallmarks of peri‐implantitis in humans, the results of this study warrants future clinical research on the effect of locally applied statins on peri‐implant tissue healing in humans as adjunct treatment to surgical peri‐implantitis therapy. Furthermore, studying carriers may be of interest due to current animal studies suggesting the importance of bioavailability of statins and its duration (Li et al., 2016; Ma et al., 2008), affecting the antibacterial properties (Akbarzadeh et al., 2021) and anti‐inflammatory effects of statins through lower T‐cell recruitment and higher vasoreactivity (Jain & Ridker, 2005). Additionally, types and dose limits of locally applied statins, leading to levels of eventual uptake in the body and nearby tissues, including effects on the peri‐implant microbiota (Belibasakis & Manoil, 2021), may need analysis and verification. Also, since retrospective study design limits finding of pre‐diabetic patients, potentially affecting peri‐implant bone loss (Alshahrani et al., 2020), a prospective study design for the effect of statins on peri‐implant bone levels of such patients may be of interest.

## CONFLICT OF INTEREST

The authors declare no conflict of interest.

## AUTHOR CONTRIBUTIONS

Behzad Bahrami‐Hessari contributed to the concept and design, acquisition of data, analysis and interpretation of data and drafted the manuscript. Leif Jansson contributed to conception and design, analysis and interpretation of data, statistical analysis and drafted the manuscript. Both authors gave final approval and agreed to be accountable for all aspects of the work.

## Data Availability

The data that support the findings of this study are available from Folktandvården Stockholms län AB, Region Stockholm, Sweden. Restrictions apply to the availability of these data, which were used with permission of local managers for this study. Data are available from the authors or research managers of the above‐mentioned organisation with the permission of Folktandvården Stockholms län AB.
